# Oligonucleotide Array and VITEK Matrix-Assisted Laser Desorption Ionization-Time of Flight Mass Spectrometry in Species Identification of Blood Yeast Isolates

**DOI:** 10.3389/fmicb.2018.00051

**Published:** 2018-01-26

**Authors:** Ming-Chi Li, Tsung C. Chang, Hung-Mo Chen, Chi-Jung Wu, Shu-Li Su, Susan S.-J. Lee, Po-Lin Chen, Nan-Yao Lee, Ching-Chi Lee, Chia-Wen Li, Ling-Shan Syue, Wen-Chien Ko

**Affiliations:** ^1^Department of Internal Medicine, National Cheng Kung University Hospital, College of Medicine, National Cheng Kung University, Tainan, Taiwan; ^2^Center for Infection Control, National Cheng Kung University Hospital, College of Medicine, National Cheng Kung University, Tainan, Taiwan; ^3^Department of Medical Laboratory Science and Biotechnology, College of Medicine, National Cheng Kung University, Tainan, Taiwan; ^4^Department of Pathology, National Cheng Kung University Hospital, College of Medicine, National Cheng Kung University, Tainan, Taiwan; ^5^National Institute of Infectious Diseases and Vaccinology, National Health Research Institutes, Tainan, Taiwan; ^6^Division of Infectious Diseases, Department of Internal Medicine, Kaohsiung Veterans General Hospital, Kaohsiung, Taiwan; ^7^Faculty of Medicine, School of Medicine, National Yang-Ming University, Taipei, Taiwan; ^8^Department of Medicine, College of Medicine, National Cheng Kung University, Tainan, Taiwan; ^9^Department of Internal Medicine, Madou Sin-Lau Hospital, Tainan, Taiwan

**Keywords:** oligonucleotide array, MALDI-TOF, vitek ms, candidemia, *Candida*, yeast

## Abstract

We evaluated matrix-assisted laser desorption ionization time-of-flight mass spectrometry using VITEK MS (IVD database) and an oligonucleotide array based on the internal transcribed spacer-1 (ITS-1) and ITS-2 sequences of rRNA genes for the identification of *Candida* spp. from blood cultures. Five-hundred and twelve consecutive bloodstream yeast isolates were collected daily and initially identified by the phenotypic automated method (VITEK YBC or VITEK2 YST card). Inconsistent results were confirmed by D1-D2 region of 28S rRNA genes and ITSs. Excluding two unidentified yeast isolates, the oligonucleotide array and VITEK MS correctly identified 99.6% (508) and 96.9% (494) of 510 yeast isolates, respectively. The oligonucleotide array and VITEK MS demonstrated high correct identification rates for four major *Candida* species (*C. albicans* 100%, 98.4%; *C. glabrata* 100%, 100%; *C. parapsilosis* 100%, 93.3%; *C. tropicalis* 100%, 97.3%), but lower correct identification rates for other *Candida* species (91.7 and 87.5%, respectively). In conclusion, the identification performance of the oligonucleotide array is comparable to that of VITEK MS, and can serve as a supplemental tool for the identification of *Candida* species.

## Introduction

Candidemia remains one of the most important invasive fungal infections, is associated with a high mortality rate (up to 30%), and is a significant healthcare burden (Hassan et al., 2009). Since a delay in the initiation of antifungal therapy for candidemia increases the risk of mortality (Garey et al., 2006), timely antifungal therapy for the treatment of candidiasis is essential. Nevertheless, the antifungal susceptibility patterns vary among different *Candida* species (Hajjeh et al., 2004), and precise and rapid identification of *Candida* species is still a challenge in clinical practice.

Matrix-assisted laser desorption ionization time-of-flight mass spectrometry (MALDI-TOF MS) has recently been introduced into clinical laboratory diagnosis as a tool for rapid identification of microorganisms, including *Candida* species (Marklein et al., 2009; Stevenson et al., 2010; Dhiman et al., 2011; Tan et al., 2012; Buchan and Ledeboer, 2013; Westblade et al., 2013). Moreover, an oligonucleotide array based on the internal transcribed spacer-1 (ITS-1) and ITS-2 sequences of rRNA genes was developed for diagnosis of invasive fungal infections and species identification (Hsiao et al., 2005; Leaw et al., 2007; Hsiue et al., 2010; Kuo et al., 2012; Li et al., 2016). The array was able to identify 77 species of clinically relevant yeasts, and the analytic procedure was completed within 8 hours. Similar to MALDI-TOF MS, the array showed superiority to conventional identification methods for *Candida* species found in blood cultures (Hsiue et al., 2010). Thus, both MALDI-TOF MS and the oligonucleotide array can be used in clinical care.

VITEK MS, a MALDI-TOF MS system, is currently used in clinical microbiology laboratories, but its identification accuracy may be unsatisfactory for some *Candida* species (Westblade et al., 2013), which may suggest that a supplemental diagnostic tool is needed. The performance of the oligonucleotide assay for species identification of candidemic isolates has not been compared to that of VITEK MS. Therefore, we analyzed yeast isolates from blood cultures and compared the identification performance of these two tools.

## Materials and Methods

### Yeast Isolates

Five-hundred and twelve yeasts isolates were collected from blood cultures from 2011 to 2014 at the National Cheng Kung University Hospital, a medical center in southern Taiwan. All blood samples were incubated in BACTEC FX (Becton, Dickinson and Company, Sparks, MD, United States), and the laboratory yeast isolates were identified by the VITEK Yeast Biochemical Card (YBC) before 2012 and VITEK 2 YST ID Card (bioMérieux, Inc., Hazelwood, MO, United States) after 2012. Morphological identification and germ tube tests were also used when necessary. Pure colonies of those isolates were further analyzed by the commercial VITEK MALDI-TOF MS system (bioMérieux; hereinafter named VITEK MS) and oligonucleotide arrays.

### Oligonucleotide Array

The species-specific probes of the oligonucleotide array were designed from the ITS-1 and ITS-2 regions of the rRNA genes. The design process and sequences of the individual probes were published previously (Leaw et al., 2007). The genomic DNA of yeast colonies was extracted, and the ITS regions were amplified by fungus specific universal primers ITS1 (5′-DIG-TCCGTAGGTGAACCTGCGG-3′) and ITS4 (5′-DIG-TCCTCCGCTTATTGATATGC-3′). The amplified PCR products amplified encompassed ITS1, ITS2, and partial regions of the 18S and 28S rRNA genes. The PCR products were further hybridized with species-specific probes as previously described (Leaw et al., 2007).

### MALDI-TOF MS

For the VITEK MS analysis, each yeast isolate was applied directly onto a target slide composed of 48-spots and was lysed with 0.5 μl of 25% formic acid (Product no. 411072, bioMérieux). After drying, 1 μl of matrix solution (Product no. 411071, bioMérieux) was applied to the spots prior to VITEK MS analysis (Iriart et al., 2012). Using VITEK MS (MS-ID version 2.0 knowledge base clinical use or *in vitro* devices [IVD]), the identification scheme produced a confidence value for each microorganism. When an organism with a confidence value of 60.0–99.9 was found, the identification result was retained and confirmed. For every isolate without a confirmed identification, the sample preparation was repeated again. If the repeated analysis still showed no matching species with adequate confidence values, the identification result was recorded as “no matched species in the database.” When the result showed “bad spectrum during acquisition,” the analysis was repeated. If the result remained “bad spectrum during acquisition,” these isolates were submitted to an extraction protocol with 70% ethanol and 70% formic acid. In brief, the collected colony was suspended in a 2 ml tube containing 900 μl of 70% ethanol. After centrifugation, the supernatant was discarded and then 40 μl of 70% formic acid was added. After complete resuspension, 40 μl of 100% acetonitrile was added, and the tube was centrifuged for at least 2 min. Then, spot immediately 1 μl of the supernatant on a target slide and allow the spot to dry completely. After air drying, 1 μl of α-cyano-4-hydroxycinnamic acid matrix solution was applied to the target plate and dried at room temperature prior to mass spectrometry analysis, as recommended by the manufacture.

### Analysis of Discrepant Specimens

For the isolates with discrepant identification among the VITEK card, oligonucleotide array, and VITEK MS, the D1-D2 region of the 28S rRNA genes and ITS region of each isolate were amplified by PCR, sequenced, and then compared with sequences in public databases using the BLAST algorithm. A threshold of ≥99% sequence identity was applied. The fungus-specific primers, ITS1 (5′-TCCGTAGGT GAACCTGCGG-3′) and ITS4 (5′-TCCTCCGCTTATTGATATGCC-3′), were used to amplify the ITS region (White et al., 1990). For D1-D2 region amplification, the primers NL1 (5′-GCATATCAATAAGCGGAGGAAAAG-3′) and NL4 (5′-GGTCCGTGTTTCA AGACGG-3′) were used (Kurtzman and Robnett, 1997). PCR products were sequenced on a model 377 sequencing system (Applied Biosystems, Taipei, Taiwan), and the results were regarded as the final identification in this study. To provide information on the identification performance for *Candida* species, we calculated the accurate identification rate of the oligonucleotide array and VITEK MS after categorizing *Candida* isolates as *C. albicans*, *C. glabrata*, *C. parapsilosis*, *C. tropicalis*, and other *Candida* species. Only an accurate identification to the species level was regarded as a correct identification. The identification results of VITEK MS were categorized into “correct identification,” “incorrect identification,” and “no matched species in the database.”

## Results

Of the 512 yeast isolates, *C. albicans, C. tropicalis, C. glabrata*, and *C. parapsilosis* accounted for 49.4% (*n* = 253), 21.5% (*n* = 110), 11.7% (*n* = 60), and 11.7% (*n* = 60), respectively. Three isolates were identified as *Lodderomyces elongisporus* (two isolates) and *Cryptococcus curvatus* (one isolate), according to the sequencing of the ITS region and 28S rRNA genes. Twenty-four isolates were composed of several uncommon *Candida* species. Two isolates were regarded as unidentified since their sequence identities according to the ITS and 28SrRNA sequences were less than 99%.

With the exclusion of the two unidentified isolates, 510 yeast isolates were further analyzed. The accuracy of the oligonucleotide array and VITEK MS results are listed in **Table 1**. The oligonucleotide array only misidentified two isolates of *C. nivariensis* as *C. glabrata* and correctly identified 99.6% (508/510) of yeast isolates. This correct identification rate was significantly higher than that of the VITEK MS (96.9%, 494/510; *P* = 0.001 by Fisher’s exact test). Of the 16 isolates unidentified by VITEK MS, 8 isolates were incorrectly identified, and 8 had no matched species in the database.

**Table 1:** Identification of yeast species by the oligonucleotide array and MALDI-TOF MS (VITEK MS).

Yeast species (Isolate no.)	Oligonucleotide array	VITEK MS
*Candida albicans* (253)	*C. albicans* (253)	*C. albicans* (249)
		*C. tropicalis* (1)
		*C. glabrata* (2)
		No matched species in database (1)
*Candida glabrata* (60)	*C. glabrata* (60)	*C. glabrata* (60)
*Candida parapsilosis* (60)	*C. parapsilosis* (60)	*C. parapsilosis* (56)
		*C. glabrata* (1)
		*C. pulcherima* (1)
		*Penicillium camemberti* (1)
		No matched species in database (1)
*Candida tropicalis* (110)	*C. tropicalis* (110)	*C. tropicalis* (107)
		No matched species in database (3)
*Candida dubliniensis* (3)	*C. dubliniensis* (3)	*C. dubliniensis* (2)
		*C. albicans* (1)
*Candida guilliermondii* (8)	*C. guilliermondii* (8)	*C. guilliermondii* (8)
*Candida haemulonii* (4)	*C. haemulonii* (4)	*C. haemulonii* (4)
*Candida krusei* (5)	*C. krusei* (5)	*C. krusei* (5)
*Candida nivariensis* (2)	*C. glabrata* (2)	No matched species in database (2)
*Candida pelliculosa* (1)	*C. pelliculosa* (1)	*C. pelliculosa* (1)
*Candida rugosa* (1)	*C. rugosa* (1)	*C. rugosa* (1)
*Cryptococcus curvatus* (1)	*Cryptococcus curvatus* (1)	*Cryptococcus curvatus* (1)
*Lodderomyces elongisporus* (2)	*L. elongisporus* (2)	*C. silvicola* (1)
		No matched species in database (1)
Unidentified yeast species (2)	Unidentified (2)	*C. guilliermondii* (1)
		No matched species in database (1)


Twenty-six isolates with discrepant identification among the oligonucleotide array, VITEK MS, and VITEK card are listed in **Table 2**. The oligonucleotide array identified all but two isolates of *C. nivariensis*. Using VITEK MS, two *C. albicans* isolates were misidentified as *C. glabrata* and one as *C. tropicalis.* No misidentification was found using VITEK MS for the species identification of *C. glabrata*. VITEK MS identified three *C. parapsilosis* isolates as *C. glabrata*, *C. pulcherima*, or *Penicillium camemberti*. Among the *C. tropicalis* isolates with discrepant identification, three *C. tropicalis* isolates had no matched species in the database. There was one *L. elongisporus* isolate misidentified as *C. silvicola* by VITEK MS.

**Table 2:** Twenty-six yeast isolates with discrepant identification by the oligonucleotide array, MALDI-TOF MS (VITEK MS) and biochemical identification (VITEK card).

Yeast species	Serial number	Oligonucleotide array	VITEK MS	VITEK card
*Candida albicans*	1	*C. albicans*	*C. glabrata*	*C. albicans*
	2	*C. albicans*	*C. albicans*	*C. parapsilosis*
	3	*C. albicans*	*C. tropicalis*	*C. albicans*
	4	*C. albicans*	*C. glabrata*	*C. albicans*
	5	*C. albicans*	No matched species in database	*C. albicans*
	6	*C. albicans*	*C. albicans*	*C. tropicalis*
	7	*C. albicans*	*C. albicans*	*C. glabrata*
	8	*C. albicans*	*C. albicans*	*C. dubliniensis*
*Candida dubliniensis*	1	*C. dubliniensis*	*C. dubliniensis*	*C. albicans*
	2	*C. dubliniensis*	*C. albicans*	*C. dubliniensis*
*Candida glabrata*	1	*C. glabrata*	*C. glabrata*	*C. parapsilosis*
*Candida nivariensis*	1	*C. glabrata*	No matched species in database	*C. glabrata*
	2	*C. glabrata*	No matched species in database	*C. glabrata*
*Candida parapsilosis*	1	*C. parapsilosis*	*C. glabrata*	*C. parapsilosis*
	2	*C. parapsilosis*	*C. pulcherima*	*C. parapsilosis*
	3	*C. parapsilosis*	*Penicillium camemberti*	*C. parapsilosis*
	4	*C. parapsilosis*	No matched species in database	*C. parapsilosis*
	5	*C. parapsilosis*	*C. parapsilosis*	*C. famata*
*Candida tropicalis*	1	*C. tropicalis*	No matched species in database	*C. tropicalis*
	2	*C. tropicalis*	No matched species in database	*C. tropicalis*
	3	*C. tropicalis*	No matched species in database	*C.* species
*Cryptococcus curvatus*	1	*Cryptococcus curvatus*	*Cryptococcus curvatus*	*C. albicans*
*Lodderomyces elongisporus*	1	*L. elongisporus*	*C. silvicola*	*C. parapsilosis*
	2	*L. elongisporus*	No matched species in database	*C. parapsilosis*
Unidentified yeast	1	Unidentified	*C. guilliermondii*	*Candida* species
	2	Unidentified	No matched species in database	*Candida* species


**FIGURE 1:**
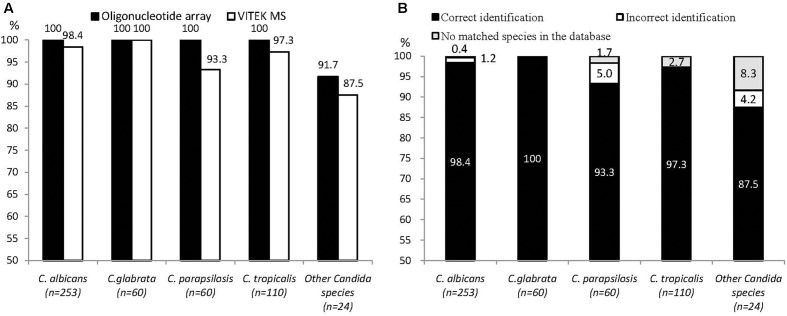
**(A)** Proportions of correct identification of the oligonucleotide array and VITEK MS in *Candida* species. **(B)** Identification results of VITEK MS in *Candida* species by proportion.

To precisely evaluate the identification performance of the oligonucleotide array and VITEK MS for *Candida* species, we excluded two unidentified yeasts and three non-*Candida* isolates (i.e., *L. elongisporus* and *C. curvatus*). The proportions of accurate identifications of *C. albicans*, *C. glabrata*, *C. parapsilosis*, *C. tropicalis*, and other *Candida* species by the oligonucleotide array and VITEK MS are shown in **Figure 1A**. The correct identification rates of *C. albicans* and *C. glabrata* by both identification tools were more than 98%. For *C. parapsilosis* and *C. tropicalis*, the correct identification rate by the oligonucleotide array was higher than that of VITEK MS (*C. parapsilosis* 100%, 93.3%; *C. tropicalis* 100%, 97.3%). For other *Candida* species, a similar trend (91.7% versus 87.5%) was noted, and lower correct identification rates were found for both methods. As for the identification results of VITEK MS, three (5%) isolates of *C. parapsilosis* and one of (4.2%) the other *Candida* species were incorrectly identified. There were no matched species in the database for two (8.3%) isolates of the other *Candida* species and three (2.7%) *C. tropicalis* isolates (**Figure 1B**).

## Discussion

Antifungal susceptibilities differ in different *Candida* species. For example, up to 13.3 and 21.4% of clinical *C. tropicalis* isolates are not susceptible to fluconazole and voriconazole, respectively (Huang et al., 2014). Accurate identification of *Candida* species would guide clinicians to optimize antifungal therapy. In this study, we collected more than 500 yeast isolates from blood to assess the performance of two identification tools. By the oligonucleotide array and VITEK MS, 99.6% (508) and 96.9% (494) of 510 yeast isolates were accurately identified, respectively. Several isolates were misidentified at the genus level by VITEK MS. By the oligonucleotide array, only two *C. nivariensis* isolates were misidentified as *C. glabrata.* These results were expected because there was no *C. nivariensis*-specific probe in the oligonucleotide array. All of the clinical isolates of four common candidemic species, *C. albicans, C. glabrata, C. parapsilosis*, and *C. tropicalis* were correctly identified by the oligonucleotide array, suggesting that the array is a potential tool for use in clinical microbiology.

In the present study, *C. albicans* accounted for less than 50% of candidemic isolates and non-*albicans Candida* isolates outweighed *C. albicans*, as noted in previous reports (Puig-Asensio et al., 2014; Hii et al., 2015). Since non-*albicans Candida* species are less susceptible to azoles (Huang et al., 2014), echinocandin therapy is preferred due to the documented therapeutic efficacy (Pappas et al., 2016). However, increasing use of echinocandins has undoubtedly provoked the emergence of echinocandin resistance (Arendrup and Perlin, 2014). Therefore, species identification is essential for the management for candidemia. MALDI-TOF MS was shown to be able to accurately identify more than 95% of yeast isolates in earlier reports (Sendid et al., 2013; Westblade et al., 2013; Hamprecht et al., 2014; Wang et al., 2016), and we found that 3.1% of yeast isolates were misidentified or could not be identified to the genus level. Jamal et al. (2014) reported that the accurate identification rates of two MALDI-TOF MS systems, VITEK MS and Bruker Biotyper MS, were 93.0% (175/188) and 92.6% (174/188), respectively (Jamal et al., 2014), highlighting the need for a performance improvement of the MALDI-TOF MS system.

Though the VITEK 2 system is popularly used in clinical microbiology laboratories, its performance is not optimal (Kim et al., 2014; Karabıçak et al., 2015; Posteraro et al., 2015). *Candida auris*, an emerging multidrug resistant pathogen causing healthcare-associated infections (Schelenz et al., 2016), was misidentified as *C. haemulonii* by the VITEK 2 system (Kathuria et al., 2015). Additionally, *L. elongisporus*, reported to cause bloodstream infections and infective endocarditis (Lockhart et al., 2008; Daveson and Woods, 2012), was misidentified as *C. parapsilosis* in this study, which may mislead the clinical judgment of physicians. Though MALDI-TOF MS was recently introduced in clinical practice, the accuracy of yeast identification by MALDI-TOF MS varies from 84 to 99% (Stevenson et al., 2010; Mancini et al., 2013), depending on the system and species tested. These findings imply the need for an alternative identification method in microbiology laboratories, in addition to traditional biochemical methods and MALDI-TOF MS. With a high accuracy rate in species identification and low cost (6.62 US dollars every assay), the oligonucleotide array may serve as an alternative yeast identification tool.

There were several limitations of this study. First, the yeast isolates were collected from a single medical center, and therefore, the results may not be generalized to other areas. However, these isolates were collected on a daily basis, which reflects real world practice. The hundreds of *Candida* isolates in this study may still provide useful information for reference. Second, there are at least two commercial MALDI-TOF MS systems, and we only compared the VITEK MS system with the oligonucleotide array. These results may not apply to the MALDI Biotyper CA System, though with respect to yeast identification, the diagnostic performance of Biotyper MS has been found to be comparable to (Hamprecht et al., 2014; Jamal et al., 2014) or slightly better than (Mancini et al., 2013; Wang et al., 2016) VITEK MS. In addition, the database of VITEK MS that we used in this study was IVD, not the VITEK MS database for research only (RUO). According to previous studies, there were some discrepant identification results between these two databases (Chao et al., 2014; Grenfell et al., 2016). Third, the identification results of several isolates were “bad spectrum during acquisition,” even after repeated testing by VITEK MS. Our initial extraction method recommended by the manufacturer for the VITEK MS IVD database used formic acid. This method was also used in several reports studying the performance of VITEK MS (Iriart et al., 2012; Won et al., 2013; Chao et al., 2014; Wang et al., 2016). For these isolates with the result of “bad spectrum,” extraction protocol with 70% ethanol and 70% formic acid may be required if on-target lysis is unsuccessful. Additionally, some *Candida* species were not included in the VITEK MS database, resulting in underestimating the performance of VITEK MS. With the information of uncommon clinical *Candida* species, the updated version of VITEK MS may improve its identification accuracy. Finally, the numbers of less common *Candida* species in this study were limited, and further studies with more isolates would be warranted.

## Conclusion

The performance of the oligonucleotide array for the identification of yeasts is comparable to that of the VITEK MS, especially for common *Candida* species in clinical practice. The reliability makes the array a promising supplemental tool for species identification of yeast isolates in blood.

## Ethics Statement

This study was approved by the Institutional Review Board of National Cheng Kung University Hospital (B-ER-103-111).

## Author Contributions

M-CL, TC, and W-CK designed the study. H-MC, C-JW, S-LS, and SL performed the experiments and interpreted the data. P-LC, N-YL, C-CL, C-WL, and L-SS helped with acquisition and data analysis. H-MC and TC: contributed critical reagents and analysis tools. M-CL and W-CK wrote and revised the manuscript. M-CL, TC, H-MC, C-JW, S-LS, SL, P-LC, N-YL, C-CL, C-WL, L-SS, and W-CK: agree to all of the aspects of the work and approved of the final work.

## Conflict of Interest Statement

The authors declare that the research was conducted in the absence of any commercial or financial relationships that could be construed as a potential conflict of interest.
